# Distinct Differences in Emotional Recognition According to Severity of Psychotic Symptoms in Early-Stage Schizophrenia

**DOI:** 10.3389/fpsyt.2019.00564

**Published:** 2019-08-12

**Authors:** Seunghee Won, Won Kee Lee, Sung-Wan Kim, Jung Jin Kim, Bong Ju Lee, Je-Chun Yu, Kyu Young Lee, Seung-Hwan Lee, Seung-Hyun Kim, Shi Hyun Kang, Euitae Kim, Young-Chul Chung

**Affiliations:** ^1^Department of Psychiatry, School of Medicine, Kyungpook National University, Daegu, South Korea; ^2^Medical Research Collaboration Center, School of Medicine, Kyungpook National University, Daegu, South Korea; ^3^Department of Psychiatry, Chonnam National University Medical School, Gwangju, South Korea; ^4^Department of Psychiatry, The Catholic University of Korea, Seoul St. Mary’s Hospital, Seoul, South Korea; ^5^Department of Psychiatry, Inje University Haeundae Paik Hospital, Inje University College of Medicine, Busan, South Korea; ^6^Department of Psychiatry, Eulji University School of Medicine, Eulji University Hospital, Daejeon, South Korea; ^7^Department of Psychiatry, Eulji University School of Medicine, Eulji General Hospital, Seoul, South Korea; ^8^Department of Psychiatry, Inje University College of Medicine, Goyang, South Korea; ^9^Department of Psychiatry, Korea University College of Medicine, Guro Hospital, Seoul, South Korea; ^10^Department of Psychiatry, Seoul National University Hospital, Seoul, South Korea; ^11^Department of Neuropsychiatry, Seoul National University Bundang Hospital, Gyeonggi-do, South Korea; ^12^Department of Psychiatry, Chonbuk National University Medical School, Jeonju, South Korea; ^13^Research Institute of Clinical Medicine of Chonbuk National University–Biomedical Research Institute of Chonbuk National University Hospital, Jeonju, South Korea

**Keywords:** schizophrenia, early stage, facial affect recognition, psychotic symptom, severity

## Abstract

Patients with schizophrenia are characterized by deficits in their ability to identify facial expressions of emotion, which are associated with impaired social and occupational function. An understanding of the deficits of facial affect recognition (FAR) early in the course of the illness can improve early intervention efforts to ameliorate potential functional deterioration. This study aimed to investigate the characteristics and correlations between psychotic symptoms and FAR deficits in patients with early-stage schizophrenia using data from the Korean Early Psychosis Cohort Study. Patients with schizophrenia were divided into three groups: 1) severely and markedly ill (n = 112), 2) moderately ill (n = 96), and 3) mildly ill (n = 115). These groups were compared with age- and sex-matched healthy controls. The FAR test was developed using Korean emotional faces from the Korean Facial Expressions of Emotion database. Error rates, correct response times, and nonresponse rates of each subset were calculated. Several psychopathology assessments were also performed. There were significantly more deficits associated with the recognition of anger (p < 0.01), fear (p < 0.01), and contempt (p < 0.01) in the three patient groups than in the healthy control group. In the severely and markedly ill states, all emotions apart from surprise had impaired error rates (p < 0.01 for all analyses). The error rates for happiness, sadness, disgust, surprise, and neutral faces were not significantly different between mildly ill patients and healthy controls. All emotions, except for sadness, had significantly more delayed correct response times in all patient groups than in the healthy controls (p < 0.01 for all analyses). The severity of psychotic symptoms was positively correlated with the happiness and neutral error rates, and depression was positively correlated with the happiness error rates. General social function was negatively correlated with the error rates for happiness, sadness, fear, disgust, and surprise. Overall, our results show that the severity of psychosis and clinical symptoms leads to distinct differences in certain emotions of patients with early-stage schizophrenia. It is considered that these specific emotional characteristics will help deepen our understanding of schizophrenia and contribute to early intervention and recovery of social function in patients with schizophrenia.

## Introduction

Schizophrenia is a chronic disorder that leads to disability in a number of clinical aspects, such as social functioning (1, 2). These disabilities are essential features and key diagnostic criteria of schizophrenia. Facial affect recognition (FAR) is a complex function involving the cortical and limbic systems and provides an indispensable source of information during face-to-face communication (3). Thus, a crucial component of successful personal interactions is to rapidly perceive facial expressions and correctly infer the internal states they convey. Facial expression misinterpretation in patients with schizophrenia generates a feeling of confusion, which triggers communication failure (4) and leads to more problems in interpersonal skills, work performance, social functioning, and independent living (5–7).

FAR has consistently been shown to be impaired in patients with schizophrenia. The impairment is present during the first episode (8), in patients with chronic schizophrenia (9), in prodromal states of psychosis (10), and in individuals at high familial risk for schizophrenia (11). Similar findings were reported for bipolar disorder (12). Thus, impairments in FAR may represent a possible endophenotype that is related to the genetic risk for and development of psychosis (13). FAR may also represent an enduring deficit and trait marker of psychosis (14).

There are often major changes in the psychosocial functioning of patients with schizophrenia within the first 3 years of onset even though the decline in function tends to plateau thereafter (15). Therefore, the first 3 years of this disorder have been described as a critical period that determines the recovery of social function, future course, and prognosis of the patient. In particular, research examining FAR deficits in people in the early stages of the illness is of critical importance. If these impairments are present early in life, they will hamper the acquisition of socially competent behaviors and ultimately alter the developmental trajectory of that individual. Patients with schizophrenia in the acute stages of the illness demonstrated a specific affect recognition deficit, but patients with chronic schizophrenia demonstrated a general face processing deficit (16, 17). Others have reported that these deficits are stable over time (14, 18). The pattern most frequently observed is that of intact recognition of positive expressions (i.e., happiness) and impaired recognition of negative expressions (i.e., anger, fear, sadness, and disgust) (19, 20). However, few studies have compared early-onset psychosis (schizophrenia) or first-episode psychosis (schizophrenia) with chronic psychosis. To date, there has only been one meta-analysis that evaluated early-onset psychosis, including schizophrenia (21). Studies that included patients with a heterogeneous diagnosis of early-onset psychosis were excluded from the analysis (21). Of the 12 studies that were analyzed in the meta-analysis, only eight included patients with schizophrenia, and of these, there were only three studies that used at least six types of specific emotions (8, 22, 23). The meta-analysis demonstrated that, in addition to general emotional recognition deficits in patients with early psychosis or first-episode psychosis, the severity of recognition deficits differed for specific emotions. Accordingly, when compared to healthy controls (HCs), large effect sizes appeared for disgust, fear, and surprise, and medium effect sizes appeared for sadness and happiness. However, there were no differences in the effect sizes for anger and neutral facial expressions. The fact that specific emotions showed differences in the extent of the recognition deficit suggested the possibility that deficits in classifying certain emotions may also be influenced by the severity of symptoms. However, all of the patients with schizophrenia in the aforementioned studies were in remission or stable, making it difficult to analyze whether symptom severity affected the extent of recognition deficits for certain emotions or whether there was any correlation between symptoms and emotional recognition deficits. In addition, the sample sizes were not very large (12–50 patients per study), which led to variation in the results depending on the characteristics of the tests and the patients.

In this study, we used data from the Korean Early Psychosis Cohort Study (KEPS) to examine if the severity of psychotic symptoms affects FAR deficits for specific emotions in patients with early-stage schizophrenia. We also analyzed possible correlations between these deficits and several psychopathologies.

## Materials and Methods

### Study Design

This study analyzed data from the KEPS, which is a naturalistic long-term prospective cohort study of patients with first-episode psychosis who were recruited from the Korean population. There are currently 11 university hospitals and one national mental health hospital participating in the KEPS. The KEPS sample consists of patients with early psychosis aged 18–45 years. Patients were defined as having early psychosis when they had received their first psychiatric treatment (outpatient or inpatient) within the last 2 years and is further divided into early stabilized patients (patients who received at least 4 consecutive weeks of antipsychotic medication with no change in dose within the last 2 months) and first-onset patients (patients who received less than 4 weeks of consecutive antipsychotic medication after the initial onset). All of the patients in the KEPS met the Diagnostic and Statistical Manual of Mental Disorders, Fifth Edition (DSM-5) (2) criteria for schizophrenia, schizophreniform disorder, schizoaffective disorder, delusional disorder, brief psychotic disorder, or other specified schizophrenia spectrum and psychotic disorders, including attenuated psychosis syndrome. Follow-up assessments were conducted at 2, 6, 9, and 12 months and then biannually through the third and fourth years. Early psychotic symptoms can be diagnosed as various disorders and can change with the clinical course of the illness, and diagnostic stability is regularly investigated using dimensional diagnosis of the DSM-5 and the Mini International Neuropsychiatric Interview (24), which is administered at baseline (registration) and 6 months, 1 year, and 2 years later. Our previously published paper provides details on the study design, methods, and subject inclusion/exclusion criteria for the KEPS (25).

For this study, we first collected data from 495 patients with early psychosis who were registered in the KEPS between January 2015 and July 2018. Patients were excluded for the following reasons: screening failure (28 patients), changed diagnosis (96 patients), no FAR test (40 patients), and incomplete data (eight patients). A total of 323 patients (134 male, 189 female) with schizophrenia or schizophreniform disorder were included for participation in the analysis. To evaluate the influence of symptom severity on FAR, we divided subjects into three groups based on their scores on the Positive and Negative Syndrome Scale (PANSS) (26, 27). Leucht et al. (28) compared the PANSS scores to ratings of the Clinical Global Impressions-Scale (CGI-S) (29). According to the CGI-S, mildly ill, moderately ill, markedly ill, and severely ill patients corresponded to PANSS total scores of 58, 75, 95, and 116, respectively. Based on these criteria, we divided patients into three groups: 1) the severely and markedly ill (SM) group (112 patients; 36 severely ill patients and 76 markedly ill patients), 2) the moderately ill (Mo) group (96 patients), and the mildly ill (Mi) group (115 patients). We used the information from 62 age- and sex-matched individuals (29 male, 33 female) for the HC group. HC data were stored at one research site from January 2013 to July 2018. Participants in the HC group did not have a personal or familial history of any DSM-IV axis I or II disorders (30) and were recruited through local advertisement. The Korean version of the Structured Clinical Interview for DSM-IV Axis I Disorders (31) was administered to all participants to confirm their diagnostic eligibility. Participants in the HC group had to be normo-thymic, which was defined as a score <8 on the Korean version of the Montgomery-Åsberg Depression Rating Scale (32) and a score <6 on the Korean version of the Young Mania Rating Scale (33). HC participants also had to be nonpsychotic, which was defined as a score ≤30 on the Brief Psychiatric Rating Scale (34). Additional exclusion criteria for the participants included head trauma, neurologic disorders, alcohol or substance abuse, mental retardation (intelligence quotient <70) as measured by the Wechsler Adult Intelligence Scale (35), and serious medical conditions. All subjects received an explanation of the research aims and the use of data and provided their written consent before participating. This study was approved by the Ethics Committee of the Chonbuk National University Hospital (approval number CUH-2014-11-002) and other participating hospitals.

### Assessment Tools

#### Psychopathology

The severity of psychotic symptoms was assessed using the PANSS and CGI. The PANSS typically consists of positive, negative, and general psychopathology subscales; however, in this study, we used a classification and scoring system that was standardized in Korea (36) and based on the 5-factor model proposed by Lindenmayer et al. (37). The 5-factor model for the PANSS (Positive, Negative, Cognitive/Disorganization, Excitement, and Depression/Anxiety subscales) has been recently recommended rather than some of the original PANSS subscales (38). We also used the Calgary Depression Scale for Schizophrenia (CDSS) (39, 40) to assess depression and the Social and Occupational Functioning Assessment Scale (SOFAS) (2) to measure general social functioning.

#### Facial Affect Recognition

To assess FAR, we modified the facial affect labeling task (41) to develop the facial emotion recognition test. This is a forced-choice emotional identification task in which eight facial expressions (happiness, sadness, anger, fear, contempt, disgust, surprise, and neutral) are presented on a computer screen. Face stimuli were acquired from the valid and reliable photographs of the Korean Facial Expressions of Emotion (KOFEE) database (42), with an established set of photographs based on characteristic facial configurations by Ekamn and Friesen (43, 44). Out of 15 actors in total (seven males, eight females), four males and four females conveying all eight emotions with higher accuracy and shorter response time were selected for the actual test. Next, two male and two female actors with high accuracy were selected for the practice session (see **Supplement** for more information). Subjects were informed of the names of the eight specific emotions that would be shown and were instructed to indicate their response by using the mouse to press the button on the screen that corresponded to the emotion that was being conveyed. Subjects saw the face and responded as quickly as possible. The pictures were displayed randomly within one block (a total of 16 pictures of facial emotions with one male and one female face for each emotion). All subjects first participated in two practice blocks. After confirming that the subjects had thoroughly understood the procedure, the actual test was performed over four blocks (a total of 64 trials). The participants were allowed a short rest between blocks. Before, during, and after this task, the participants remained in a stable emotional state. Face stimuli appeared during 750 ms, and the intertrial interval was 4,500 ms (3,000 ms of reaction time plus 1,500 ms of feedback time).

### Statistical Analysis

The primary outcomes of this study were accuracy and response time (mean correct response time) for each emotion. For accuracy, we calculated commission error rates (mean error rate) and omission error rates (mean nonresponse rate). The secondary outcomes were the correlation coefficients between recognition deficits for each emotion and several psychopathologies.

All analyses were conducted using SAS 9.4 (Copyright 2002–2012 by SAS Institute Inc., Cary, NC, USA). Values of p < 0.05 were regarded as significant. For the demographic and clinical data, we performed analysis of variance (ANOVA) to examine group differences for numerical data. Following the ANOVA, we used Tukey-Kramer’s post-hoc correction to compare the groups. We used the chi-square test for categorical data. Analyses of covariance and Tukey-Kramer’s post-hoc comparisons were performed to analyze the accuracy (error rate, nonresponse rate) and correct response time of the facial emotion recognition test results. The peak age for schizophrenia is 10–25 years in men and 25–35 years in women (45); therefore, it is considered that the difficulties in performing academic work after disease onset are caused by differences in education levels. Because of this difference, educational level was used as a covariate when analyzing the accuracy and response time. In patients, Pearson’s correlations were performed to detect the relationship between psychopathology and performance on the facial emotion recognition test within patient groups. To compare the extent and patterns of emotional recognition deficits between the three patient groups and the HC group, we calculated the effect size for each emotion. Effect sizes (Cohen’s d) were calculated based on the average standard deviation from the two means. A value of 0.2 indicated a small effect size, 0.5 indicated a medium effect size, and 0.8 indicated a large effect size (46).

## Results

### Subject Disposition and Clinical Characteristics

The demographic characteristics and clinical features of patients are summarized in **Table 1**. There were no significant differences in mean age between the SM group (28.07 ± 8.15 years), Mo group (27.81 ± 8.34 years), Mi group (27.47 ± 7.39 years), and HC group (29.31 ± 5.31 years). There were also no significant differences among the groups regarding sex, marital status, or monthly income. However, education was significantly higher in the control group than in the patient groups (χ² = 23.01, p < 0.01). The duration of untreated psychosis (DUP), the ratio of patients on antipsychotics at the time of registration, and mean antipsychotics dosage (converted to chlorpromazine equivalents) were not significantly different among the three patient groups.

**Table 1:** Demographic and clinical data of the subjects.

Variables	Severely and markedly ill state^a^ (n = 112)	Moderately ill state^b^ (n = 96)	Mildly ill state^c^ (n = 115)	Healthy controls (n = 62)	F/χ²	P	Post hoc*
**Mean age**	28.07 ± 8.15	27.81 ± 8.34	27.47 ± 7.39	29.31 ± 5.31	0.56	0.640	
**Sex, male (n,%)**	44, 39.27	44, 45.83	46, 40.00	29, 46.77	1.67	0.645^†^	
**Education** **High school or less (n, %)** **College or higher (n, %)**	53, 47.32 59, 52.68	36, 37.50 60, 62.50	45, 39.13 70, 60.87	7, 11.29 55, 88.71	23.01	0.000^†^	
**Marital state** **Unmarried (n, %)** **Married (n, %)**	97, 86.61 15, 13.39	79, 82.29 17, 62.50	93, 80.87 22, 19.13	44, 70.97 18, 29.03	6.50	0.090^†^	
**Monthly income (%) (10,000 won)** **<150** **150–350** **>350**	23, 20.54 55, 49.11 34, 30.36	17, 17.90 49, 51.58 29, 30.53	16, 14.04 56, 49.12 42, 36.84	6, 9.68 31, 50.00 25, 40.32	5.29	0.508^†^	
**DUP (month)**	15.27 ± 19.35	13.24 ± 29.97	15.16 ± 27.52		0.19	0.826	
**Antipsychotics** **User ratio (n, %)** **Mean dosage** **^‡^** ** (mg)**	59, 52.82 400.14 ± 245.78	48, 50.00 348.96 ± 266.95	46, 40.00 355.14 ± 262.33		4.04 0.55	0.133 0.576	
**CGI-S**	4.38 ± 1.04	3.62 ± 1.06	2.89 ± 1.06		57.35	0.000	a > b > c
**PANSS** **Total** **Positive** **Negative** **Cognitive/Disorganization** **Excitement** **Depression/Anxiety**	93.58 ± 17.91 13.35 ± 3.33 16.92 ± 4.68 19.92 ± 5.30 15.47 ± 4.56 13.38 ± 4.03	66.86 ± 4.87 9.48 ± 2.23 12.51 ± 3.51 13.96 ± 3.43 10.35 ± 2.81 10.36 ± 2.81	48.19 ± 7.48 6.78 ± 2.06 8.55 ± 2.55 10.32 ± 2.35 7.17 ± 2.04 7.70 ± 2.39		426.14 180.40 146.65 174.57 158.74 91.75	0.000 0.000 0.000 0.000 0.000 0.000	a > b > c a > b > c a > b> c a > b > c a > b > c a > b > c
**CDSS**	7.50 ± 5.63	5.45 ± 4.47	2.62 ± 2.47		36.69	0.000	a > b > c
**SOFAS**	49.61 ± 13.69	57.65 ± 9.41	62.81 ± 12.48		34.11	0.000	a < b < c

p value was calculated using ANOVA. ^†^p value was calculated using chi-square test. *Analysis of variance and Tukey-Kramer’s post-hoc comparison were performed. n, number; DUP, duration of untreated psychosis; ^‡^chlorpromazine equivalents. CGI-S, Clinical Global Impression-Severity; PANSS, Positive and Negative Syndrome Scale; CDSS, Calgary Depression Scale for Schizophrenia; SOFAS, Social and Occupational Functioning Assessment Scale. ^a^denoted a severely & markedly ill, ^b^denoted a moderately ill, ^c^denoted a mildly ill stage groups.

The PANSS total scores and CGI-S scores, which are used to assess psychosis severity, in the SM group were significantly higher than were the scores in the Mo and Mi groups, and the scores in the Mo group were significantly higher than were the scores in the Mi group (F = 426.14, p < 0.01; F = 57.35, p < 0.01, respectively). The PANSS subscale scores, including the scores on the Positive, Negative, Cognitive/Disorganization, Excitement, and Depression/Anxiety subscales were also significantly different among the patient groups (F = 180.40, p < 0.01; F = 146.65, p < 0.01; F = 174.57, p < 0.01; F = 158.74, p < 0.01; F = 91.75, p < 0.01, respectively). The CDSS scores were significantly different among the three groups (F = 36.69, p < 0.01). The SOFAS scores were also significantly different among all three groups (F = 34.11, p < 0.01). For all analyses, the patients in the SM group had the highest scores, followed by the patients in the Mo group, and finally, the patients in the Mi group.

In the HC group, the scores on the Brief Psychiatric Rating Scale (19.33 ± 1.99), Korean version of the Young Mania Rating Scale (0.36 ± 0.78), and Korean version of the Montgomery-Åsberg Depression Rating Scale (1.61 ± 2.20) indicated that psychotic and mood symptoms were all within the normal range.

### Primary Outcomes

#### Commission Error Rates

Commission error rates for each emotion are summarized in **Table 2**. Compared to the HC group, the SM group showed significantly higher error rates for all emotional faces except surprise; the Mo group showed significantly higher error rates for sadness, anger, fear, contempt, and disgust; and the Mi group showed significantly higher error rates for anger, fear, and contempt. Patients in the SM group showed significantly higher error rates for surprise than did the patients in the Mi group (F = 4.64, p = 0.003); however, there were no significant differences in the error rate for surprise between each patient group and the HC group.

**Table 2:** Commission error rates (percent of error trials) in the facial emotion recognition test: comparison of each patient group by disease severity in patients with schizophrenia and healthy controls.

Variables	Severely and markedly ill state^a^ (n = 112)	Moderately ill state^b^ (n = 96)	Mildly ill state^c^ (n = 115)	Healthy controls^d^ (n = 62)	F	*p*	Post hoc*
**Happiness**	0.09 ± 0.01	0.05 ± 0.01	0.03 ± 0.01	0.02 ± 0.01	9.17	0.000	a > bcd
**Sadness**	0.26 ± 0.02	0.25 ± 0.02	0.18 ± 0.02	0.18 ± 0.03	4.04	0.008	ab > cd
**Anger**	0.43 ± 0.03	0.39 ± 0.03	0.40 ± 0.03	0.26 ± 0.04	4.87	0.003	abc > d
**Fear**	0.75 ± 0.02	0.69 ± 0.03	0.67 ± 0.02	0.53 ± 0.03	9.37	0.000	abc > d
**Contempt**	0.35 ± 0.03	0.27 ± 0.03	0.22 ± 0.03	0.09 ± 0.04	10.17	0.000	a > cd bc > d
**Disgust**	0.56 ± 0.03	0.57 ± 0.03	0.49 ± 0.03	0.46 ± 0.04	3.41	0.018	ab > d
**Surprise**	0.12 ± 0.01	0.09 ± 0.01	0.05 ± 0.01	0.11 ± 0.02	4.64	0.003	a > c
**Neutral**	0.09 ± 0.01	0.08 ± 0.02	0.06 ± 0.01	0.03 ± 0.02	2.79	0.040	a > d

Estimated the marginal means over a balanced population. p value was the result of analysis of covariance (ANCOVA) adjusted for education. *Post-hoc comparisons using Tukey-Kramer’s method following a significant ANCOVA. ^a^denoted a severely & markedly ill, ^b^denoted a moderately ill ^c^denoted a midly ill states, ^d^denoted a healthy control groups.

The effect sizes for the FAR deficit relative to the HC group are summarized in **Table 3**. In the SM group, contempt and fear showed large effect sizes; anger, happiness, sadness, and neutral faces showed medium effect sizes; and disgust showed a small effect size. In the Mo group, contempt showed a large effect size; fear, anger, sadness, neutral, and happiness showed medium effect sizes; and disgust showed a small effect size. In the Mi group, contempt, anger, and fear showed medium effect sizes; neutral, happiness, and surprise showed small effect sizes.

**Table 3:** The effect size (Cohen’s *d*) of the error rates in the facial emotion recognition test: comparison of each patient group and healthy controls.

Variables	Severely and markedly ill state - Healthy controls	Moderately ill state - Healthy controls	Mildly ill state - Healthy controls
**Happiness**	-0.655	-0.462	-0.243
**Sadness**	-0.584	-0.493	-0.164
**Anger**	-0.734	-0.574	-0.665
**Fear**	-0.921	-0.674	-0.582
**Contempt**	-1.049	-0.809	-0.684
**Disgust**	-0.419	-0.438	-0.131
**Surprise**	-0.091	0.070	0.433
**Neutral**	-0.563	-0.486	-0.322

#### Omission Error Rates

The patients in the SM group showed significantly higher nonresponse rates for all emotional faces than did the patients in the HC group. The patients in the Mo group showed significantly higher nonresponse rates for fear, contempt, and disgust than did the patients in the HC group. The patients in the Mi group showed significantly higher nonresponse rates for fear than did the patients in the HC group (**Table 4**).

**Table 4:** Omission error rates (percent of nonresponse trials) in the facial emotion recognition test: comparison of each patient group and healthy controls.

Variables	Severely and markedly ill state^a^ (n = 112)	Moderately ill state^b^ (n = 96)	Mildly ill state^c^ (n = 115)	Healthy controls^d^ (n = 62)	F	*p*	Post hoc*
**Happiness**	0.049 ± 0.006	0.021 ± 0.007	0.010 ± 0.006	0.007 ± 0.009	7.94	0.000	a > bcd
**Sadness**	0.092 ± 0.011	0.056 ± 0.011	0.052 ± 0.010	0.014 ± 0.015	6.34	0.000	a > cd
**Anger**	0.115 ± 0.011	0.069 ± 0.012	0.048 ± 0.011	0.023 ± 0.015	9.58	0.000	a > cd
**Fear**	0.125 ± 0.013	0.069 ± 0.013	0.051 ± 0.012	0.010 ± 0.017	11.12	0.000	a > b > c > d
**Contempt**	0.107 ± 0.012	0.081 ± 0.013	0.030 ± 0.012	0.007 ± 0.016	11.40	0.000	ab > cd
**Disgust**	0.171 ± 0.016	0.108 ± 0.017	0.103 ± 0.015	0.010 ± 0.021	13.07	0.000	a > bcd bc > d
**Surprise**	0.059 ± 0.009	0.043 ± 0.009	0.015 ± 0.008	0.007 ± 0.012	6.67	0.000	a > cd
**Neutral**	0.069 ± 0.011	0.040 ± 0.011	0.020 ± 0.010	0.006 ± 0.014	5.47	0.000	a > cd

Estimated marginal means over a balanced population. p value was the result of analysis of covariance (ANCOVA) adjusted for education. *Post-hoc comparisons using Tukey-Kramer’s method following a significant ANCOVA. ^a^denoted a severely & markedly ill, ^b^denoted a moderately ill, ^c^denoted a mildly ill states, ^d^denoted a healthy control groups.

#### Response Time

Apart from sadness, all emotions showed slower correct response times in all patient groups compared to the HC group (p < 0.01 for all analyses). There were no significant differences in the correct response times for anger, fear, contempt, disgust, surprise, and neutral among the three patient groups. However, the correct response time for sadness was slower in the SM group than in the HC group (**Table 5**).

**Table 5:** Correct response time (mm second) in the facial emotion recognition test: comparison of each patient group and healthy controls.

Variables	Severely and markedly ill state^a^ (n = 112)	Moderately ill state^b^ (n = 96)	Mildly ill state^c^ (n = 115)	Healthy controls^d^ (n = 62)	F	*p*	Post hoc*
**Happiness**	1,679.42 ± 30.31	1,557.12 ± 26.02	1,541.61 ± 29.70	1,281.83 ± 41.37	19.62	0.000	a > bc > d
**Sadness**	2,107.11 ± 105.10	1,864.01 ± 113.90	1,875.64 ± 103.00	1,598.04 ± 143.57	2.75	0.042	a > d
**Anger**	2,063.41 ± 46.52	2,057.71 ± 48.95	2,061.61 ± 44.28	1,666.43 ± 59.98	11.83	0.000	abc > d
**Fear**	2,151.56 ± 56.20	2,133.59 ± 56.15	2,120.31 ± 51.64	1,808.94 ± 65.06	6.69	0.000	abc > d
**Contempt**	1,898.33 ± 47.76	1,804.43 ± 50.44	1,817.70 ± 44.61	1,386.99 ± 61.72	15.43	0.000	abc > d
**Disgust**	2,252.63 ± 52.73	2,284.44 ± 54.76	2,319.41 ± 49.33	1,986.41 ± 66.74	5.83	0.000	abc > d
**Surprise**	1,775.08 ± 33.59	1,703.97 ± 35.86	1,706.79 ± 32.78	1,464.84 ± 45.66	10.15	0.000	abc > d
**Neutral**	1,594.33 ± 32.29	1,572.17 ± 34.14	1,536.03 ± 31.20	1,216.79 ± 43.48	18.42	0.000	abc > d

Estimated marginal means over a balanced population. p value was the result of analysis of covariance (ANCOVA) adjusted for education. *Post-hoc comparisons using Tukey-Kramer’s method following a significant ANCOVA.^a^denoted a severely & markedly ill, ^b^denoted a moderately ill, ^c^denoted a mildly ill states, ^d^denoted a healthy control groups.

### Secondary Outcomes

The PANSS total scores, which indicate the severity of psychosis, were positively correlated with the error rates for happiness (r = 0.226, p < 0.01), surprise (r = 0.212, p < 0.01), sadness (r = 0.166, p < 0.01), and contempt (r = 0.128, p < 0.05). The CGI-S scores, which also indicate the severity of psychosis, were positively correlated with the error rates for happiness (r = 0.185, p < 0.01) and surprise (r = 0.158, p < 0.01). Among the PANSS subscales, the Positive subscale score was positively correlated with the error rates for surprise (r = 0.159, p < 0.01) and happiness (r = 0.118, p < 0.05). The PANSS Negative subscale score was positively correlated with the error rates for surprise (r = 0.253, p < 0.01), happiness (r = 0.243, p < 0.01), sadness (r = 0.179, p < 0.01), contempt (r = 0.122, p < 0.05), and anger (r = 0.118, p < 0.05). The PANSS Cognitive/Disorganization subscale score was positively correlated with the error rates for happiness (r = 0.247, p < 0.01), sadness (r = 0.168, p < 0.01), contempt (r = 0.157, p < 0.01), surprise (r = 0.177, p < 0.01), neutral (r = 0.136, p < 0.05), and fear (r = 0.111, p < 0.05). The PANSS Excitement subscale score was positively correlated with the error rates for happiness (r = 0.190, p < 0.01), surprise (r = 0.183, p < 0.01), neutral (r = 0.166, p < 0.05), and contempt (r = 0.134, p < 0.05). The PANSS Depression/Anxiety subscale score was not significantly correlated with any emotion; however, there was a significant positive correlation between the CDSS score and the error rates for happiness (r = 0.111, p < 0.05). Finally, the SOFAS score was negatively correlated with the error rates for sadness (r = -0.117, p < 0.01), fear (r = -0.151, p < 0.01), happiness (r = -0.125, p < 0.05), surprise (r = -0.117, p < 0.05), and disgust (r = -0.112, p < 0.05) (**Table 6**).

**Table 6:** Correlations between psychopathology and error rates in the facial recognition test within the schizophrenia groups (n = 323).

Variables	PANSS Positive	PANSS Negative	PANSS Cognitive/ Disorganization	PANSS Excitement	PANSS Depression/ Anxiety	PANSS Total	CGI-S	CDSS	SOFAS
**Happiness**	0.118*	0.243^†^	0.247^†^	0.190^†^	0.099	0.226^†^	0.185^†^	0.111*	-0.125*
**Sadness**	0.105	0.179^†^	0.168^†^	0.103	0.089	0.165^†^	0.079	0.084	-0.200^†^
**Anger**	-0.018	0.118*	0.091	0.047	-0.023	0.064	-0.007	-0.016	-0.062
**Fear**	0.056	0.090	0.111*	0.080	-0.010	0.084	0.003	0.031	-0.151^†^
**Contempt**	0.093	0.122*	0.157^†^	0.134*	-0.010	0.128*	0.036	-0.031	-0.102
**Disgust**	0.065	0.091	0.085	0.088	0.034	0.085	0.076	-0.014	-0.112*
**Surprise**	0.159^†^	0.253^†^	0.177^†^	0.183^†^	0.066	0.212^†^	0.158^†^	0.034	-0.117*
**Neutral**	0.069	0.054	0.136*	0.166*	0.048	0.109	0.079	0.041	-0.087

*p < 0.05, ^†^p < 0.01. PANSS, Positive and Negative Syndrome Scale; CGI-S, Clinical Global Impression-Severity; CDSS, Calgary Depression Scale for Schizophrenia; SOFAS, Social and Occupational Functioning Assessment Scale.

## Discussion

We investigated the influence of symptom severity on FAR deficits for various emotions in patients with early-stage schizophrenia. To the best of our knowledge, this is the first study to examine, in detail, the relationship between psychotic symptoms and the characteristics of FAR deficits for specific emotions. Understanding the characteristics and patterns of emotional recognition deficits, which are known to be closely related to social function, is important for improving our understanding of symptoms in early-stage schizophrenia and plays an important role in disease prognosis and the recovery of social function.

There were no differences between the patients and controls in age, sex ratio, monthly income, or marital status, but the patient groups did show lower education levels than the HCs. The PANSS total scores and CGI-S scores, which both evaluate the overall severity of psychosis, were mutually consistent and showed significant differences among the three patient groups. Using the PANSS 5-factor model established by Leucht et al. (28), the PANSS Positive, Negative, Cognitive/Disorganization, Excitement, and Depression/Anxiety subscales showed significant differences among the three patient groups. The CDSS and SOFAS also both showed significant differences among the three patient groups. For all analyses, patients in the SM group had the highest scores, followed by patients in the Mo group, and finally, patients in the Mi group. All of these findings suggest that all patients were appropriately classified by symptom severity and general functioning.

The three patient groups showed no differences in DUP or medication (chlorpromazine equivalent dose), indicating that there was little selection bias with regard to duration of disease or medication. Typically, it is believed that patients with more severe psychotic symptoms use higher dosages of antipsychotics; however, in this study, the dosage of antipsychotics was not different among the three patient groups. This is considered to be because only a few patients with severe symptoms were included, and even patients in the acute phase do not often use higher dosages of medication initially. Additionally, drug compliance was low; only half of the patients were using medication at the time of registration in the three groups.

In this study, the relationship between accuracy of emotional recognition and severity of psychotic symptoms for specific emotions can be explained as follows. The commission error rates for happiness, sadness, anger, fear, contempt, disgust, and neutral were significantly higher in the SM group than in the HC group. As the severity of symptoms decreased, the error rates for happiness and neutral faces improved, followed by the error rates for sadness and disgust. The error rates for anger, fear, and contempt were higher in the three patient groups than in the HC group. The error rates of surprise were not significantly different between the patient groups and the HC group. In the SM group, the effect sizes for contempt, fear, anger, happiness, sadness, neutral, and disgust were high. Additionally, contempt, fear, and anger consistently showed greater deficits across all levels of symptom severity. Happiness, sadness, disgust, and neutral recognition showed decreasing effect sizes with improved symptoms. There were no deficits in surprise recognition between patients in the three groups and patients in the HC group.

There have been prior studies of various emotions in stable, first-onset patients compared to HC groups. For instance, Edwards et al. (8) reported deficits in sadness and fear but not in happiness, anger, disgust, surprise, or neutral (contempt was not studied). Leung et al. (22) reported significant differences in surprise, fear, and disgust but not in anger, sadness, or happiness (contempt and neutral were not studied), whereas Comparelli et al. (23) reported differences in fear, disgust, anger, and sadness but not in happiness or surprise (contempt and neutral were not studied). Considering our results and those of prior studies, fear appears to demonstrate consistent recognition deficits across all studies, whereas happiness and neutral consistently demonstrate no deficits in patients in mild or stable condition. Anger, sadness, surprise, and disgust demonstrated inconsistent results (contempt cannot be compared across studies because it was only included in ours).

Lee et al. (47) performed a study of 55 Korean stable patients with chronic schizophrenia (mean age, 32.1 ± 8.1; years since first hospitalization 8.2 ± 5.9) using an FAR test with Korean faces and reported that the patients showed differences in sadness, fear, and anger recognition. However, there were no differences in the recognition of happiness, surprise, disgust, and neutral expressions (contempt was not studied). Even though this study was conducted with patients in chronic conditions, all results, except of those for sadness, are consistent with the results of our study. Studies comparing FAR deficits in first-episode and chronic-stage patients have reported that initial deficits are stable over time up to the chronic stage (14, 18, 22) and that deficits in the chronic phase are somewhat more generalized compared to the early stage (16, 17). The fact that our study and that of Lee et al. both showed similar FAR deficit patterns supports the theory that FAR deficits in first-episode patients are stable over time. Cross-cultural or cross-national differences in FAR deficits have been reported even in healthy individuals (48). Although the FAR deficits reported in patients with schizophrenia across all cultures share the same characteristics, it has been reported that there are differences in the FAR deficits for specific emotions (49). The fact that our results were closer to those of Lee et al. than to those from other cultures suggests that there are cultural differences in the recognition of specific emotions.

In an early study, Addington and Addington (9) found that improvements in positive and negative symptoms were not accompanied by improvements in face recognition in patients with schizophrenia. This suggests that face discrimination processing may be unrelated to disease severity. Subsequently, most studies have found a significant association between face recognition and negative symptoms but not positive symptoms (5, 50, 51). However, some of these studies also found a specific relationship between affect recognition and positive symptoms, such as bizarre behavior (52), thought disorder (53), and overall positive symptoms (54). Thus, investigations of the relationship between affect recognition and specific symptoms have yielded mixed findings, and a more detailed research has not yet been performed. Given the diverse severity of symptoms among our patient groups, we were in a suitable position to assess the symptoms and characteristics of each emotion. Among assessments of psychotic symptom severity, the PANSS total scores were positively correlated with the error rates for happiness, surprise, sadness, and contempt; the CGI-S scores were positively correlated with the error rates for happiness and surprise. This demonstrated that FAR deficits for expressions of happiness and surprise are associated with general psychotic symptoms. Among the PANSS subscales, the Positive subscale score was positively correlated with the error rates for surprise and happiness; the Negative subscale score was positively correlated with the error rates for surprise, happiness, sadness, contempt, and anger; the Cognitive/Disorganization subscale score was positively correlated with the error rates for happiness, sadness, contempt, surprise, neutral, and fear; and the Excitement subscale score was positively correlated with the error rates for happiness, surprise, neutral, and contempt. Depression in patients with schizophrenia was associated with higher error rates for happiness recognition. Finally, there were negative correlations between general social function and the error rates for sadness, fear, happiness, surprise, and disgust.

Few reports have evaluated omission rates; therefore, we cannot evaluate our findings in the context of previous results. In our study, the nonresponse rates for all emotional faces were significantly higher in the SM group than in the HC group. As the symptoms improved, the nonresponse rates for happiness also improved, followed by contempt, neutral, surprise, sadness, and anger. The nonresponse rates for fear and disgust were higher in the patient groups than in the HC group. All patients showed delayed responses to all emotions, except for sadness, regardless of the severity of psychotic symptoms. The results for response time and omission rate indicated that patients with schizophrenia experienced difficulties in emotional information processing, which provides evidence for the reliability of our results.

Overall, the relationship between emotional recognition and clinical symptoms for specific emotions in early-stage schizophrenia can be explained as follows. First, the accuracy of all emotions and response times were impaired (except for surprise) in patients who were severely and markedly ill. Second, the error rate for happiness was positively correlated with the PANSS total score and the CGI-S, CDSS, and SOFAS scores; therefore, we believe that happiness recognition is the state marker most closely related to general symptoms and social function. Third, the error rate for the neutral expression was positively correlated with the PANSS cognitive/disorganization and PANSS Excitement subscales, suggesting that this was the most sensitive state marker for initial improvement in the acute psychotic state. Fourth, anger, fear, and contempt recognition continued to show medium to large deficits even when symptoms improved, and response rates and omission rates both showed significant differences across all three patient groups. There was no correlation between the severity of psychotic symptoms (especially anger and fear), denoting that these are likely to be schizophrenia-specific trait markers that are scarcely affected by psychotic symptom severity. Fifth, the effect size for sadness, disgust, and surprise recognition indicated a mild or lower deficit in the recognition of these emotions. Therefore, patients with schizophrenia may be able to recognize these emotions well and show a somewhat appropriate response. Sixth, there were negative correlations between general social function and sadness, fear, happiness, surprise, and disgust. Depression in patients with schizophrenia was associated with impairments in happiness recognition.

This study had a few limitations. First, as this was a cross-sectional study rather than a longitudinal one, we were unable to identify differences in the same subjects according to the states of the illness. Moreover, the aim of this study was not to assess the impact of FAR deficits on longitudinal prognosis. However, the present results will contribute toward the understanding FAR deficits and clarify potential differences in its pathogenesis according to states of early-stage schizophrenia. Further studies are required to investigate these issues. Second, although we controlled for the level of education, cognitive function was not well-controlled because only discrete variables, such as intelligence, were used. Third, our emotion recognition task has some shortcomings, including a relatively low correction rate for fear and disgust in HCs (47% and 54%, respectively).

However, the major strength of our study is the large effect size of the planned comparisons between each clinical group and the HC group. Second, to our knowledge, this is the first study comparing the symptom severity of early-stage schizophrenia for each of the eight basic emotions. This study is a step toward the elucidation of emotion recognition impairment in schizophrenia. The understanding of the interactions between emotional recognition, social cognition, and social functioning in schizophrenia should be a goal for future research in the field of early intervention.

## Conclusion

This study used data from the KEPS to examine the correlation between symptom severity and the extent of emotional recognition deficits for different emotions in patients with early-stage schizophrenia. We divided patients into three groups based on the severity of psychotic symptoms (SM, Mo, and Mi groups) and tested the recognition of facial expressions by Korean actors. The results showed deficits in all emotions apart from surprise in the SM group. There were deficits in the recognition of anger, fear, and contempt across all patient groups. There were no differences in the error rates for happiness, sadness, disgust, and surprise between the Mi and HC groups. The correct response times for all emotions, except for sadness, were significantly more delayed in patients in the three symptom groups than in the HC group. The severity of psychotic symptoms was positively correlated with happiness and the neutral error rates, and depression was positively correlated with the happiness error rates. General social function showed negative correlations with the error rates for happiness, sadness, fear, disgust, and surprise. Our results are similar to those of a previous study that examined patients with chronic schizophrenia in Korea, suggesting that some emotional recognition deficits are stable over time and that there are cultural differences for certain emotions.

## Author Contributions

SW and Y-CC conceptualized the study. SW, S-WK, JJK, BJB, J-CY, KYL, S-HL, S-HK, SHK, EK and Y-CC performed the study and acquired data. SW and WKL conducted statistical analyses. SW and Y-CC analyzed and interpreted the data. SW drafted the manuscript. SW and Y-CC critically revised the manuscript. Y-CC received the grant. All authors approved the final manuscript.

## Funding

This study was funded under contract/grant number HM14C2608 from the Korean Mental Health Technology R&D Project, Ministry of Health & Welfare, South Korea.

## Conflict of Interest Statement

The authors declare that the research was conducted in the absence of any commercial or financial relationships that could be construed as a potential conflict of interest.
