# Supplementary material for: Distinct Differences in Emotional Recognition According to Severity of Psychotic Symptoms in Early-Stage Schizophrenia
Source: Front Psychiatry. 2019 Aug 12;10:564. doi: 10.3389/fpsyt.2019.00564 (PMC6699582; doi:10.3389/fpsyt.2019.00564)
Supplement: Supplementary file 1 [file DataSheet_1.pdf]

How the stimulus photographs were selected from the Korean Facial Expressions of Emotion (KOFEE) database

There are 176 photographs of 15 performers (seven male, eight female) on the KOFEE database that have passed reliability tests. Of the eight female photographs, only four conveyed all eight emotions. Four women with all eight emotions and four men in the order of highest mean reliability were selected for the actual test, and even the same person chose to be highly reliable if they had multiple pictures of one emotion. The mean reliability was calculated as the average of the eight emotional reliabilities. The subject codes and photograph numbers used in the actual test are shown in Table 1. Next, two men and two women with high mean reliability were selected for the practice session. Similarly, we selected the photographs with high reliability among the many available. Table 2 shows the subject codes and photograph numbers used in the practice session.

The use of all the photographs was approved by the original author of the KOFEE database.

Table 1. Subject codes and the numbers of the photographs used in the actual test from the KOFEE

ID/Sex	Happiness	Disgust	Anger	Sadness	Surprise	Fear	Contempt	Neutral
**DY/male**	8301	8374	8558	8491	8325	8338	8502	8295
**JM/male**	8111	8082	8092	8119	8064	8071	8129	8062
**KD/male**	9822	9880	9990	9951	9832	9850	9962	9813
**EW/male**	8952	8993	9025	9053	8964	8983	9070	8946
**BM/female**	7950	7874	7927	7956	7840	7855	7963	7835
**YJ/female**	8675	8755	8894	8841	8688	8740	8357	8671
**SJ/female**	8287	8223	8276	8300	8204	8213	8315	8197
**SR/female**	9014	9060	9122	9142	9026	9046	9155	9006

Table 2. Subject codes and the numbers of the photographs used in the practice session from the KOFEE

ID/Sex	Happiness	Disgust	Anger	Sadness	Surprise	Fear	Contempt	Neutral
**DS/male**	8950	8885	8896	8967	8858	8873	8995	8847
**HJ/male**	9519	9580	9605	9619	9542	9549	9629	9513
**DB/female**	9176	9267	9318	9337	9188	-	9361	9171
**JS/female**	8576	8622	8633	8647	8588	-	8660	8567
